# The complete mitochondrial genome and phylogenetic analysis of *Aoria bowringii* (Baly, 1860)

**DOI:** 10.1080/23802359.2025.2609545

**Published:** 2025-12-30

**Authors:** Ke Zhang, Hongfei Liang, Chong Luo

**Affiliations:** a School of Life Sciences, Guizhou Normal University, Guiyang, China; b College of Teacher Education, Guizhou Normal University, Guiyang, China

**Keywords:** *Aoria bowringii*, *Cayratia japonica*, mitochondrial genome, phylogenetic analysis

## Abstract

We sequenced and annotated the complete mitochondrial genome of *Aoria bowringii*. The circular mitogenome is 17,054 bp in length and exhibits a strong A + T bias, with nucleotide composition A 47.71%, T 30.63%, C 16.00%, and G 5.67%. It contains the typical set of 37 metazoan mitochondrial genes, including 13 protein-coding genes (PCGs), 22 transfer RNA genes, and two ribosomal RNA genes, as well as a putative control region. A maximum-likelihood phylogenetic analysis based on 13 PCGs places *A. bowringii* as a highly supported sister species to its congener *Aoria nigripes*. This mitogenome provides a valuable molecular resource for future phylogenetic and population genetic studies of this insect pest.

## Introduction

**Figure 1.**
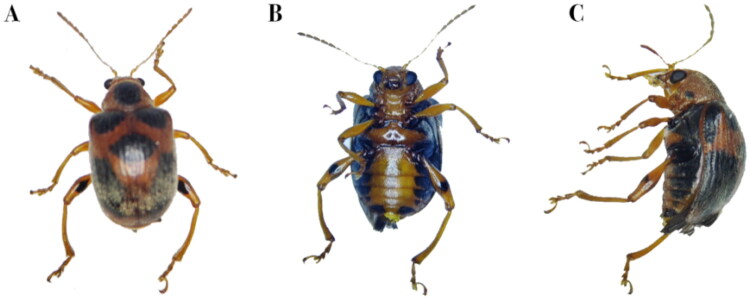
*Aoria bowringii* (Baly, 1860), female. (A) Dorsal view; (B) ventral view; (C) lateral view. Photographs were taken by Ke Zhang in the *Ampelopsis grossedentata* Germplasm Resource Nursery of Guizhou Normal University, Guiyang, Guizhou Province, China.

*Aoria bowringii* (Baly, 1860) (Coleoptera: Chrysomeloidea: Eumolpinae) is widely distributed in China, Vietnam, Cambodia, Laos, Thailand, Myanmar, Nepal, India, Indonesia, and adjacent regions (Tan et al. 2017). Adults are characterized by a small, deeply sculptured head, elongated antennae reaching approximately half of the body length, distinct black spots on the prosternum and elytra, a subspherical pronotum and densely punctate elytra with 11 striae. Legs are long and robust, and body size ranges from 5 to 6 mm in length and 2.7 to 3.8 mm in width (Figure 1).

Field investigations have demonstrated that both *A. bowringii* and *A. nigripes* pose considerable threats to the traditional Chinese medicinal plant, *Cayratia japonica* (Figure S1). Previous research has predominantly concentrated on *A. nigripes*, which is known to inflict significant damage on grapevines and other host plants. It is commonly found on both cultivated and wild grapevines, including *Parthenocissus himalayana*, *Crystal Grape*, and *Quercus* (Hua 2002; Qin et al. 2004; Zhou et al. 2015). Adult *A. nigripes* typically feed on the adaxial leaf surfaces, as well as the epidermis of branches and petioles, producing distinctive hollow feeding strips (Figure S2) (Zhou et al. 2015). In agricultural systems, chemical control of *A. nigripes* frequently involves the application of 3% acetamiprid, 4.5% beta-cypermethrin, or 2.5% bifenthrin (Wang et al. 2011). Furthermore, the complete mitochondrial genome and phylogenetic status of *A. nigripes* have been described (Yang et al. 2022). Conversely, limited research has addressed the occurrence and management of *A. bowringii* on medicinal and economically significant *Ampelopsis* species. In the present study, we report the complete mitochondrial genome sequence of *A. bowringii* and assess its phylogenetic position within the superfamily Chrysomeloidea, thereby offering a valuable genomic reference for future studies. The objective of this study was to characterize the complete mitogenome of *A. bowringii* and infer its phylogenetic position within Chrysomeloidea based on 13 mitochondrial protein-coding genes (PCGs), providing baseline information to support future biological control strategies.

## Materials and methods

Adults of *A. bowringii* were collected by hand from *Cayratia japonica* plants growing in the medicinal plant nursery of Guizhou Normal University, Guiyang City, Guizhou Province, China (E 106.6326, N 26.3848). The insect specimen was deposited at the School of Life Sciences, Guizhou Normal University (Ke Zhang; e-mail 17802927850m0@sina.cn) under voucher number GZNUZK232200101546.

Total genomic DNA was extracted from thoracic muscle tissue using an Animal Tissue Genomic DNA Extraction Kit (Meiji Yuhua, Shanghai, China) following the manufacturer’s protocol. A paired-end sequencing library with an average insert size of approximately 350 bp was constructed. Paired-end sequencing was performed on an Illumina Nova-Seq 6000 platform (San Diego, CA). Raw data were subjected to quality filtering with Cut adapt (Martin 2011) to remove adapter contamination and low-quality reads, yielding high-quality clean reads in FASTQ format for downstream analyses.

Clean reads were used to assemble the mitochondrial genome with both GetOrganelle v1.7.0+ (Jin et al. 2020) and NOVOPlasty v4.2 (Dierckxsens et al. 2017). The published mitochondrial genome of *A. nigripes* (Yang et al. 2022) was used as a reference sequence to guide assembly. Both approaches produced an identical circular mitogenome in terms of length, gene order, and gene content; minor discrepancies at the boundaries of the control region were checked and manually corrected before annotation.

The final mitogenome was annotated using MITOS2 (Bernt et al. 2013) under the invertebrate mitochondrial genetic code. Annotations were manually checked and refined using CPS tools (Huang et al. 2024). The complete annotated mitogenome of *A. bowringii* has been deposited in GenBank. Nucleotide composition was calculated for the whole mitogenome and for each PCG, tRNA, and rRNA gene. AT- and GC-skew values were computed as AT-skew = (A − T)/(A + T) and GC-skew = (G − C)/(G + C).

To determine the phylogenetic position of *A. bowringii*, we analyzed the concatenated nucleotide sequences of 13 mitochondrial PCGs from *A. bowringii* and 23 related species with complete or nearly complete mitogenomes available in GenBank. Taxa were chosen to cover representatives of Eumolpinae and other major lineages within Chrysomeloidea, while *Eutorrhynchus sisymbrii* and *Ceutorhynchus pulvinatus* were used as out-groups. Individual PCGs were aligned codon-wise using MAFFT v7 (Katoh and Standley 2013) and then concatenated into a single nucleotide matrix. The best-fit substitution model for the concatenated dataset was selected with jModelTest2 (Darriba et al. 2012) under the Akaike information criterion and identified as GTR + I + G. A maximum-likelihood tree was inferred in RAxML v8 (Stamatakis 2014) under the GTR + I + G model with 1000 bootstrap replicates.

## Results

**Figure 2.**
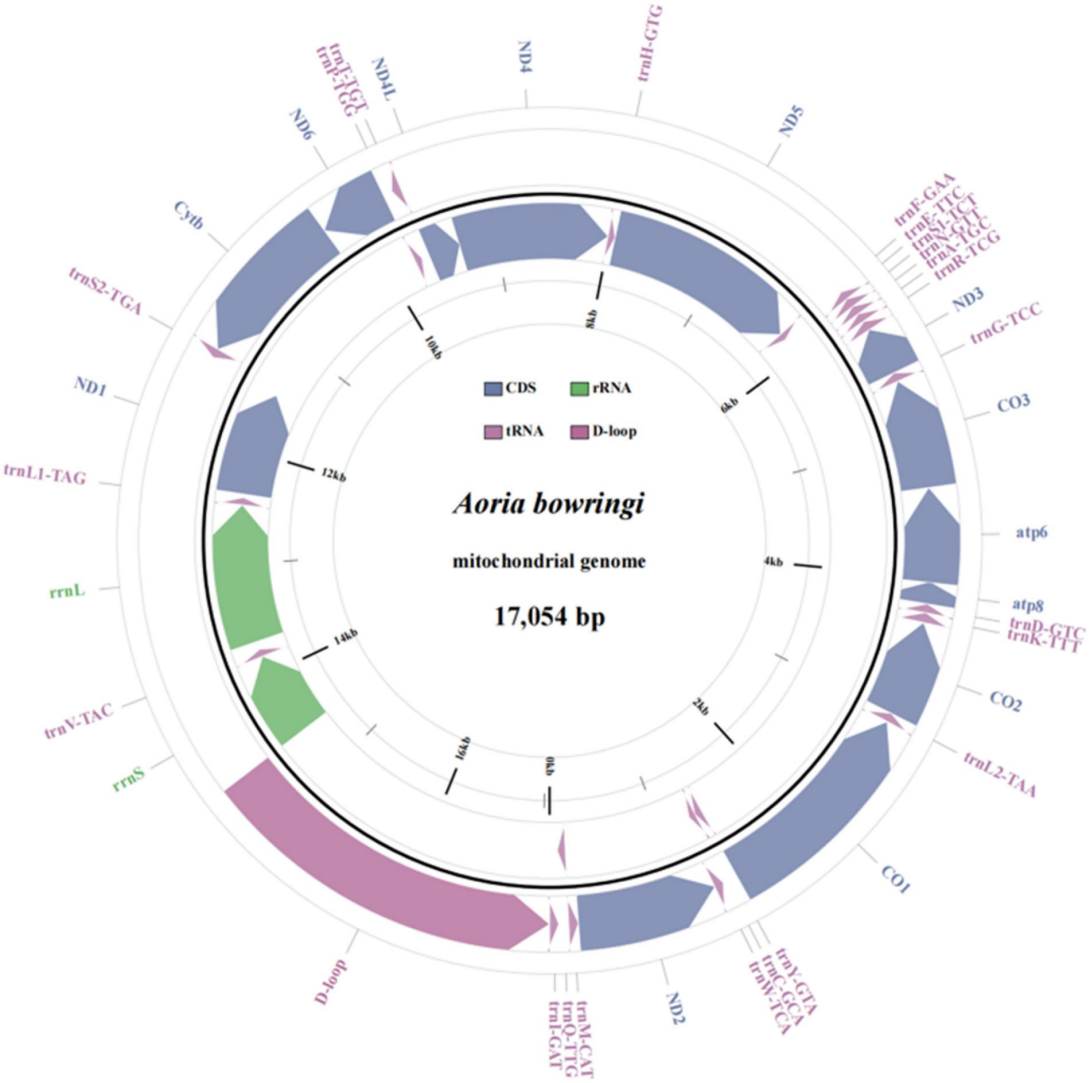
Gene map of the mitochondrial genome for *A. bowringii* (GenBank accession number: PV563566). Arrows indicate the orientation of gene transcription. Protein coding sequences (CDS), tRNA, rRNA, and D-loop are marked with different colors. The tRNAs are labeled according to their anticodons.

Illumina sequencing generated 67,666,676 raw reads. After quality filtering, 67,638,102 clean reads were retained for downstream analyses. The complete mitochondrial genome of *A. bowringii* is a circular molecule of 17,054 bp in length (GenBank accession number: PV563566). It contains the typical set of 37 metazoan mitochondrial genes, including 13 PCGs, 22 tRNA genes, and two rRNA genes (*rrnL* and *rrnS*), along with a large non-coding A + T-rich control region (Figure 2). The mean coverage was 1783× (Figure S2). The overall gene order is consistent with that of other published chrysomelid mitogenomes. The nucleotide composition is A (47.71%), T (30.63%), C (16.00%), and G (5.67%) with A + T bias (78.34%). The light strand encodes four PCGs (*ND5*, *ND4*, *ND4L*, and *ND1*) and eight tRNA genes: *trnQ*(TTG), *trnC*(GCA), *trnY*(GTA), *trnF*(GAA), *trnH*(GTG), *trnP*(TGG), *trnL1*(TAG), and *trnV*(TAC), and the heavy strand encodes the other tRNA genes. This compositional bias was also reflected in the skew values, with a positive AT-skew (0.22) and a negative GC-skew (−0.48), which suggests a preferential use of A and C over T and G on the coding strand (Table S1). The majority of PCGs use standard ATN start codons (ATG or ATT) and terminate with TAA or TAG stop codons (Table S2). TAA serves as the stop codon for *ND2*, *CO1*, *ATP8*, *ATP6*, *ND4*, *ND4L*, and *ND6*, whereas TAG is used by *Cytb* and *ND1*. Four PCGs (*CO2*, *CO3*, *ND3*, and *ND5*) end with an incomplete stop codon consisting of a single T, which is presumed to be converted into a complete TAA stop codon via post-transcriptional polyadenylation. The 22 tRNA genes range from 59 to 71 bp in length, and the rRNA genes *rrnL* and *rrnS* are 1214 bp and 739 bp long, respectively.

**Figure 3.**
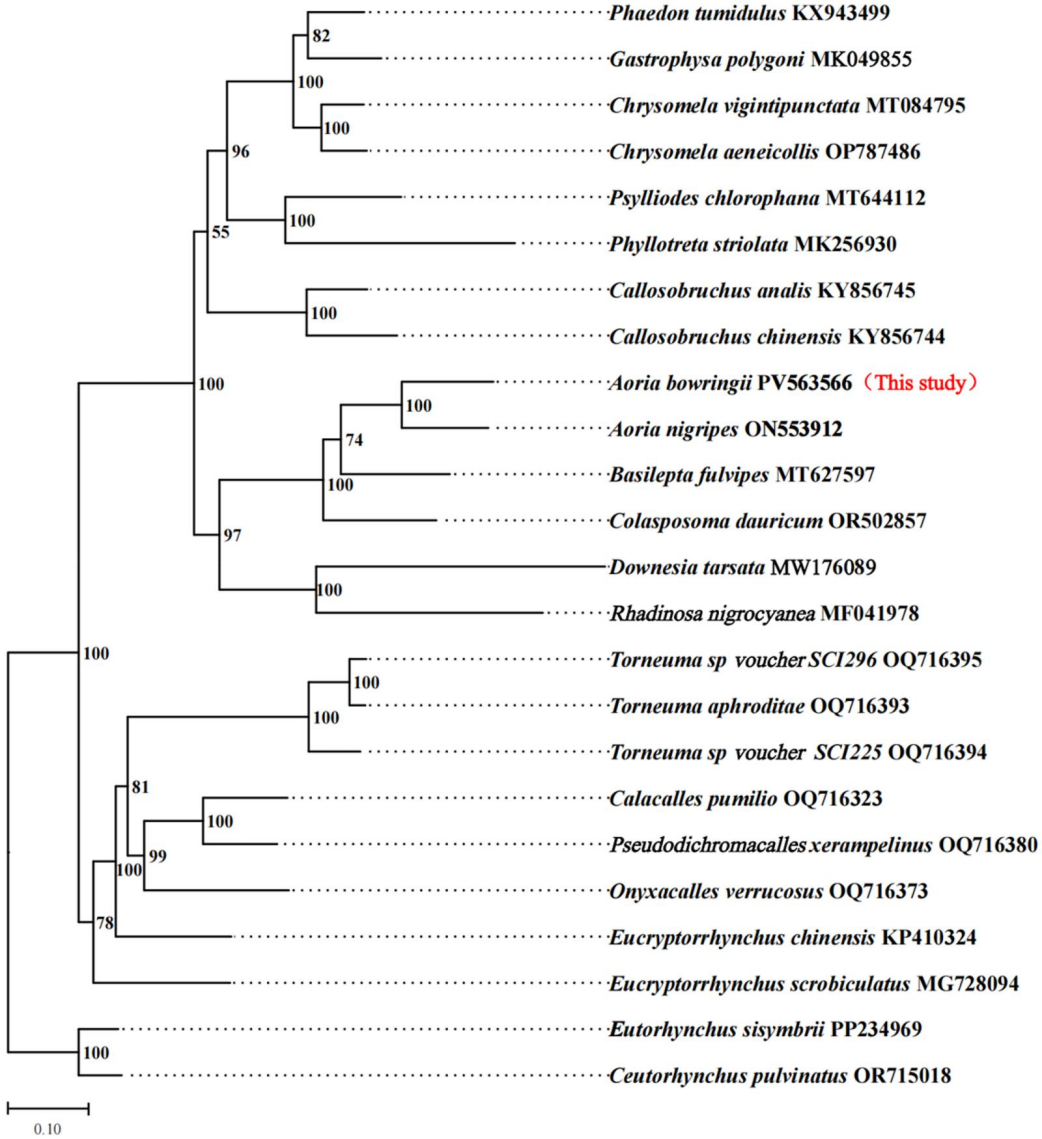
The maximum-likelihood tree, based on the complete mitogenome sequences of 24 species. GenBank accession numbers are described in the figure. Shown next to the nodes are bootstrap support values, based on 1000 replicates. The following sequences were used: *A. bowringii* (this study), *Eutorrhynchus sisymbrii* PP234969 and *Ceutorhynchus pulvinatus* OR715018 (Letsch et al. 2024), *Eucryptorrhynchus scrobiculatus* MG728094 (Lin et al. 2024), *Eucryptorrhynchus chinensis* KP410324 (Liu et al. 2016), *Onyxacalles verrucosus* OQ716373, *Pseudodichromacalles xerampelinus* OQ716380, *Torneuma sp*. voucher SCI296 OQ716395, *Torneuma sp*. voucher SCI225 OQ716394 and *Torneuma aphroditae* voucher LG461xCED0677 OQ716393 (Jiménez-García et al. 2023), *Downesia tarsata* NC_056105 (Zhang et al. 2021), *A. nigripes* NC_065028 (Yang et al. 2022), *Colasposoma dauricum* OR502857 (Li et al. 2022), *Basilepta fulvipes* MT627597 (Liu et al. 2020), *Callosobruchus analis* KY856745 and *Callosobruchus chinensis* KY856744 (Sayadi et al. 2017), *Psylliodes chlorophana* MT644112 (Zhang et al. 2016), *Phyllotreta striolata* MK256930 (Zu and Yan 2019), *Chrysomela vigintipunctata* MT084795 (Yan et al. 2020), *Chrysomela aeneicollis* OP787486 (Bracewell et al. 2023), *Phaedon tumidulus* KX943499 (Gómez-Rodríguez et al. 2015), *Rhadinosa nigrocyanea* MF041978 (Liu et al. 2024), *Calacalles pumilio* OQ716323 and *Gastrophysa polygoni* NC_045247 (unpublished).

The resulting ML tree placed *A. bowringii* as a sister taxon to *A. nigripes* with maximum bootstrap support (100%) (Figure 3). This result strongly supports its classification within the genus *Aoria* and is in accordance with traditional morphological classification. This newly reported mitogenome serves as a valuable molecular resource for future studies on the phylogeny and population genetics of the *subfamily* Eumolpinae, family Chrysomelidae.

## Discussion and conclusions

The complete mitochondrial genome of *A. bowringii* (17,054 bp), reported here for the first time, exhibits genomic features typical of Chrysomeloidea. Its gene content, gene order, codon usage, and strong A + T bias closely resemble those of its congener *A. nigripes*, highlighting the conserved nature of mitogenome architecture in this group.

The maximum-likelihood phylogeny based on 13 concatenated mitochondrial PCGs robustly places *A. bowringii* as the sister species to *A. nigripes* (bootstrap support = 100%; Figure 3), consistent with their traditional morphological classification within Eumolpinae (Tan et al. 2017). The mitogenome presented here therefore provides a reliable reference for future studies on the genetic diversity, population structure, and evolutionary history of this agriculturally important pest.

## Supplementary Material

Supplementary Figures.docx

Supplementary Table.docx

## Data Availability

The data of this study are openly accessible in GenBank of NCBI under the accession number PV563566. The associated BioProject, SRA, and BioSample numbers are PRJNA1156990, SRR30575198, and SAMN43504603, respectively.
