# Supplementary material for: The complete mitochondrial genome and phylogenetic analysis of Aoria bowringii (Baly, 1860)
Source: Mitochondrial DNA B Resour. 2025 Dec 30;11(1):205–9. doi: 10.1080/23802359.2025.2609545 (PMC12777889; doi:10.1080/23802359.2025.2609545)

**Supplementary fig. 1**.Plants of the *Causonis japonica* were damaged by *Aoria bowringii.* This photo was taken by the author Ke Zhang at *Ampelopsis. grossedentata* Germplasm Resource Nursery of Guizhou Normal University in Guiyang, Guizhou Province, China.


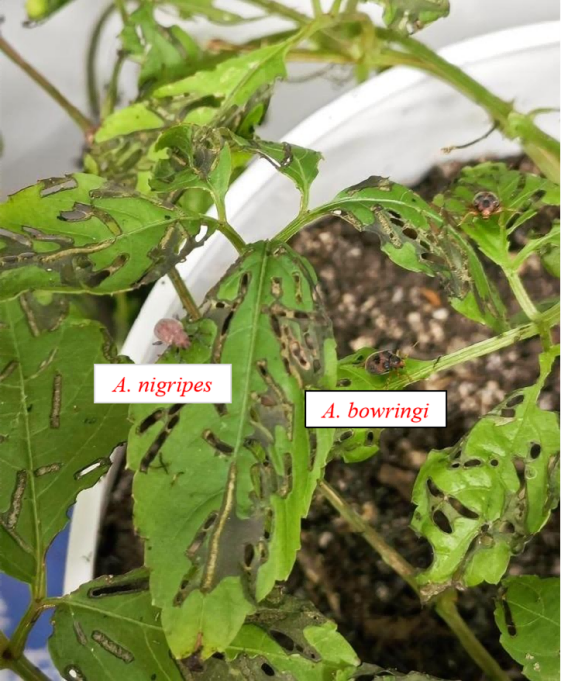


**Supplementary fig. 2.** The coverage depth of the complete mitochondrial genome of *A. bowringii.*

(1)Total genome length = 17,054 bp (2) Average depth = 1783 x

(3) Maximaldepth = 3790 x (4) Minimal depth= 193 x （at the joint）


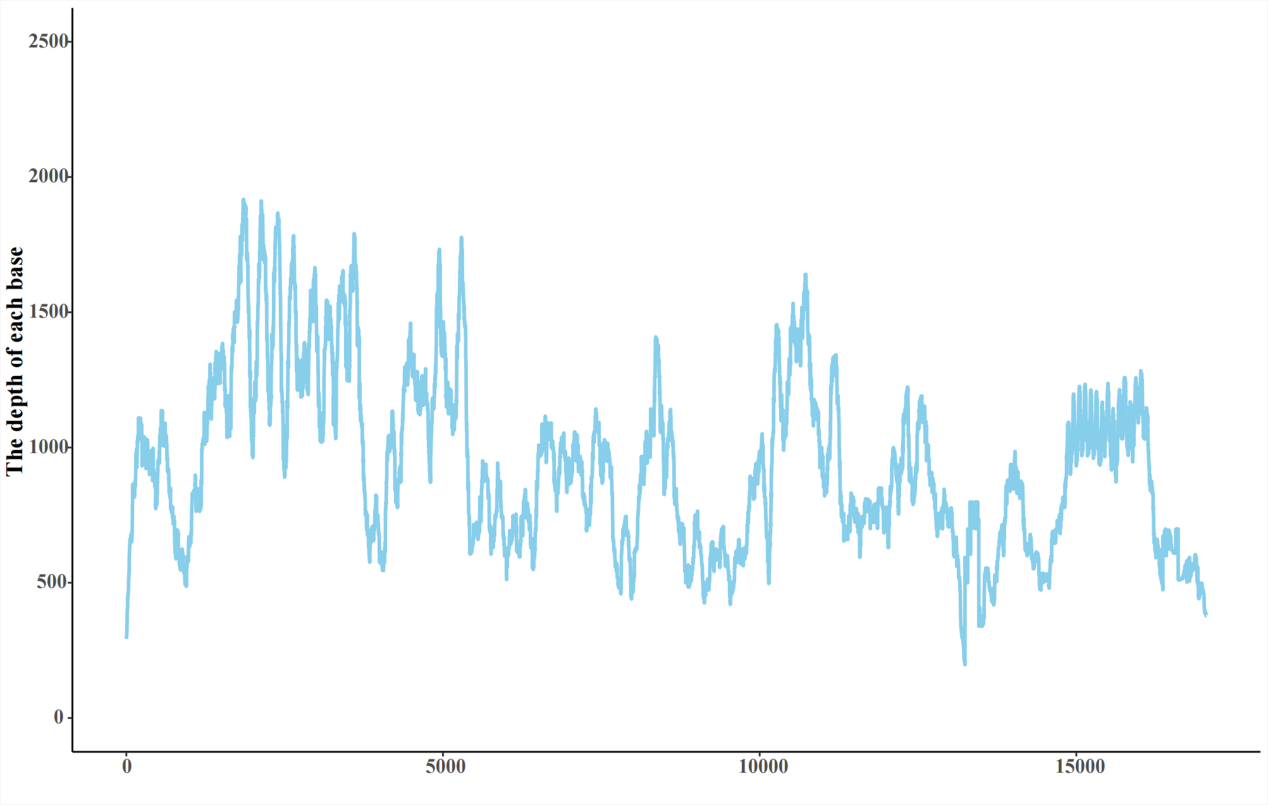

Supplement: Supplementary Figures.docx [file TMDN_A_2609545_SM5318.docx]
