# Supplementary material for: The complete mitochondrial genome and phylogenetic analysis of Aoria bowringii (Baly, 1860)
Source: Mitochondrial DNA B Resour. 2025 Dec 30;11(1):205–9. doi: 10.1080/23802359.2025.2609545 (PMC12777889; doi:10.1080/23802359.2025.2609545)
Supplement: Supplementary Table.docx [file TMDN_A_2609545_SM5317.docx]

**Table S1.** Summary of the characteristics of the mitogenome of *A. bowringii*.

| Region | Size(bp) | A(%) | T(%) | G(%) | C(%) | A+T(%) | AT-skew | GC-skew |
| --- | --- | --- | --- | --- | --- | --- | --- | --- |
| Mitogenome | 17054 | 47.71 | 30.63 | 5.67 | 16.00 | 78.34 | 0.22 | -0.48 |
| *cox1* | 1545 | 32.62 | 36.63 | 14.89 | 15.86 | 69.25 | -0.06 | -0.03 |
| *cox2* | 684 | 35.67 | 36.84 | 12.13 | 15.35 | 72.51 | -0.02 | -0.12 |
| *atp8* | 159 | 42.77 | 38.99 | 3.77 | 14.47 | 81.76 | 0.045 | -0.59 |
| *atp6* | 675 | 36.00 | 39.41 | 9.04 | 15.56 | 75.41 | -0.05 | -0.27 |
| *cox3* | 787 | 33.93 | 37.99 | 13.09 | 14.99 | 71.92 | -0.056 | -0.07 |
| *nad3* | 354 | 34.46 | 42.66 | 8.47 | 14.41 | 77.12 | -0.11 | -0.26 |
| *nad1* | 915 | 27.65 | 49.95 | 14.1 | 8.31 | 77.6 | -0.29 | 0.26 |
| *nad5* | 1719 | 29.92 | 49.71 | 12.82 | 7.55 | 79.63 | -0.25 | 0.26 |
| *nad4* | 1333 | 27.99 | 51.59 | 12.48 | 7.94 | 79.58 | -0.30 | 0.22 |
| *nad4l* | 288 | 29.51 | 51.74 | 13.54 | 5.21 | 81.25 | -0.27 | 0.44 |
| *nad6* | 507 | 44.79 | 37.01 | 5.73 | 12.47 | 81.80 | 0.10 | -0.37 |
| *cob* | 1140 | 34.47 | 38.42 | 11.23 | 15.88 | 72.89 | -0.05 | -0.17 |
| *nad2* | 1008 | 38.00 | 41.57 | 7.74 | 12.70 | 79.56 | -0.04 | -0.24 |
| *tRNAs* | 1436 | 41.25 | 37.46 | 12.11 | 9.18 | 78.71 | 0.05 | 0.13 |
| *rRNAs* | 1953 | 35.58 | 44.59 | 13.36 | 6.48 | 80.17 | -0.11 | 0.35 |
| *PCGs* | 11074 | 34.44 | 42.51 | 10.69 | 12.36 | 76.95 | -0.10 | -0.07 |

**Table S2.** Features of protein-coding genes detected in the mitochondrial genome of *A. bowringii*.

| Gene | Gene Length(bp) | A + T Content (%) | Start/Stop Codon |
| --- | --- | --- | --- |
| *nad2* | 1008 | 79.56 | ATT/TAA |
| *cox1* | 1545 | 69.26 | ATT/TAA |
| *cox2* | 684 | 72.51 | ATC/TAG |
| *atp8* | 159 | 81.76 | ATC/TAA |
| *atp6* | 675 | 75.41 | ATG/TAA |
| *cox3* | 787 | 71.92 | ATG/T-- |
| *nad3* | 354 | 77.12 | ATA/TAA |
| *nad5* | 1708 | 79.63 | ATA/T-- |
| *nad4* | 1322 | 79.58 | ATG/TA- |
| *nad4l* | 288 | 81.25 | ATG/TAA |
| *nad6* | 488 | 81.8 | ATA/TAA |
| *cob* | 1140 | 72.89 | ATG/TAG |
| *nad1* | 915 | 77.60 | ATT/TAG |
| Total | 11074 |  |  |
